# A Rare Case of Thrombotic Thrombocytopenic Purpura (TTP) With Concurrent Renal Cell Carcinoma: Diagnostic and Therapeutic Challenges

**DOI:** 10.7759/cureus.39494

**Published:** 2023-05-25

**Authors:** Amit Bhandari, Bidushi Pokhrel, Prakash R Oli, Quang Le, Bibhusan Basnet, Eric C Freitag, Archana Nayani

**Affiliations:** 1 Internal Medicine, St John's Hospital, Springfield, USA; 2 Intensive Care Unit, Hospital for Advanced Medicine and Surgery, Kathmandu, NPL; 3 Internal Medicine, Province Hospital, Surkhet, NPL; 4 Hospital Medicine, University of Missouri School of Medicine, Columbia, USA; 5 Internal Medicine, Frye Regional Medical Center, Hickory, USA; 6 Pathology and Laboratory Medicine, St. John's Hospital, Springfield, USA; 7 Hematology and Oncology, Springfield Clinic, Springfield, USA

**Keywords:** rcc, renal cell cancer, tma, thrombotic microangiopathy, ttp, thrombotic thrombocytopenic purpura

## Abstract

Thrombotic thrombocytopenic purpura (TTP) is a potentially life-threatening thrombotic microangiopathy (TMA) that needs prompt identification and treatment. Disseminated malignancy-related TMA can potentially be misdiagnosed as TTP, and patients may be inappropriately subjected to therapeutic plasma exchange (TPE) with serious implications. Likewise, the presence of a concurrent cancer diagnosis in a patient with microangiopathic hemolytic anemia and thrombocytopenia may lead to suspicion of disseminated malignancy as the cause, delaying the TPE with serious outcomes. Testing for ADAMTS13 activity is diagnostic of TTP, but the results may take time. This poses a diagnostic and therapeutic dilemma that includes weighing the benefits of TPE for treating TTP and cancer treatment. We describe a rare case of immune-mediated TTP in a patient concurrently diagnosed with metastatic renal cell cancer. To our knowledge, this is the first case of TTP reported in patients with metastatic renal cell carcinoma (RCC) in a non-treatment-naive patient.

## Introduction

Thrombotic microangiopathy (TMAs) includes a heterogeneous group of diseases characterized by microangiopathic hemolytic anemia, peripheral thrombocytopenia, and organ failure of varying severity. Primary TMAs include thrombotic thrombocytopenic purpura (TTP), caused by a severe functional deficiency of ADAMTS13, a specific von Willebrand factor cleaving protease, or hemolytic uremic syndrome (HUS), mainly caused by abnormalities of the alternate complement pathway. Secondary TMAs include several distinct causes, including Shiga toxin due to *Escherichia coli (E. coli),* pregnancy-related TMAs, HUS associated with infections unrelated to *E. coli* Shiga toxin, transplantations, autoimmune diseases, drugs, and disseminated malignancies [1-3].

Disseminated malignancy is a rare but well-known cause of TMA that can add to therapeutic and diagnostic challenges. Several disseminated cancers are associated with thrombocytopenia and hemolytic anemia. Often, a cancer diagnosis may not be clinically apparent at the time of presentation. Plasma ADAMTS13 activity levels are useful to differentiate TTP from cancer-related TMAs, but the result may take days. Therefore, patients with cancer-related TMAs may be hastily and incorrectly diagnosed with TTP and subjected to plasmapheresis, which is not beneficial. On the other hand, a concurrent cancer diagnosis in a patient with microangiopathic hemolytic anemia and thrombocytopenia may lead to suspicion of disseminated malignancy and a delay in the initiation of therapeutic plasma exchange (TPE) [4].

We describe a case of immune-mediated TTP in a patient who was concurrently diagnosed with metastatic renal cell cancer. TTP in the setting of renal cell cancer (RCC) in treatment-naive patients is extremely rare. To our knowledge, this is the first case of TTP in the setting of concurrent RCC in a treatment-naive patient. We discuss the diagnostic and therapeutic challenges faced in managing this patient and review the available literature.

## Case presentation

A 42-year-old African American man without any known medical problems presented to the emergency room (ER) with complaints of slurred speech and confusion. He had been experiencing severe dizziness, lightheadedness, and fatigue for a few weeks before presenting to the ER. He reported an intentional weight loss of about 50 pounds over the past six months with diet, exercise, and lifestyle modification. His family history was significant for sickle cell disease in his sister and colon cancer in his mother, both diagnosed at age 52. He had undergone laparotomy for a gunshot wound in the 1990s. He worked as a welder for a truck company and denied any illicit drugs but admitted to vaping and smoking legal marijuana. On the initial examination, his vitals were stable. He was found to be confused and disoriented, but otherwise, no focal neurological deficits were noted. The rest of the systematic exams were unremarkable.

His full blood count and chemistry results were remarkable for low hemoglobin of 5.8 gram/deciliter (g/dl), platelet count of 23000 per microliter, elevated reticulocyte count (6.3%), mildly elevated lactate dehydrogenase (LDH) of 549 units/liter (U/L), and low haptoglobin (<7.8 milligram/deciliter). The Coombs test was negative. The coagulation panel and disseminated intravascular coagulation (DIC) panel were negative. A comprehensive biochemical panel, including liver function tests, renal function tests, and electrolytes, was normal. The PLASMIC score was calculated at 5 (Table 1).

**Table 1:** Initial labs at the time of presentation/admission to our hospital

Labs		References range and units
Complete Blood Count		
Hemoglobin	5.8	13.0 - 18.0 G/DL
Platelet	23	150 - 350 x10'3/uL
White blood cells	12.1	4.00 - 10.80x10'3/uL
Reticulocyte count	6.3%	0.7 - 2.3 %
Haptoglobin	<7.8	30.0 - 200.0 MG/DL
Mean corpuscular volume (MCV)	75.9	78.0 - 100.0 FL
Comprehensive metabolic panel		
Sodium	142	136 - 145 MMOL/L
Potassium	3.9	3.5 - 5.1 MMOL/L
Bicarbonate	23.5	21.0 - 32.0 MMOL/L
Blood urea nitrogen	8	7 - 18 MG/DL
Serum creatinine	0.88	0.70 - 1.30 MG/DL
Glomerular filtration rate (GFR)	>90 (ml/min/1.73 m^2^)	>90 ML/MIN/1.73 M2
Blood glucose	83	74 - 106 MG/DL
Vitamin B12	440	193 - 986 PG/ML
Coagulation panel		
Prothrombin time (PT)	12.8	9.4 - 12.5 Sec
International normalized ratio (INR)	1.1	0.8-1.1
Fibrinogen	358	200 - 393 MG/DL
PLASMIC score	5	

**Figure 1:**
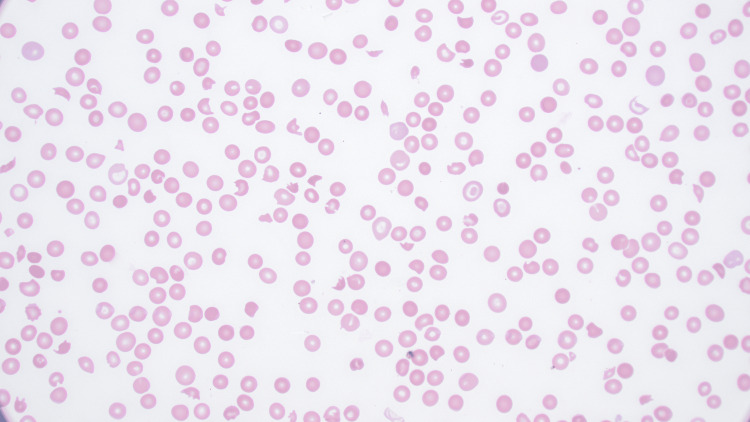
Peripheral blood smear showing scattered schistocytes (Wright’s stain, 600x)

A peripheral smear showed multiple schistocytes (Figure 1).

ADAMTS13 level was sent and awaited. He was initially admitted to the intensive care unit (ICU) with a working diagnosis of TTP in anticipation of the need for TPE.

**Figure 2:**
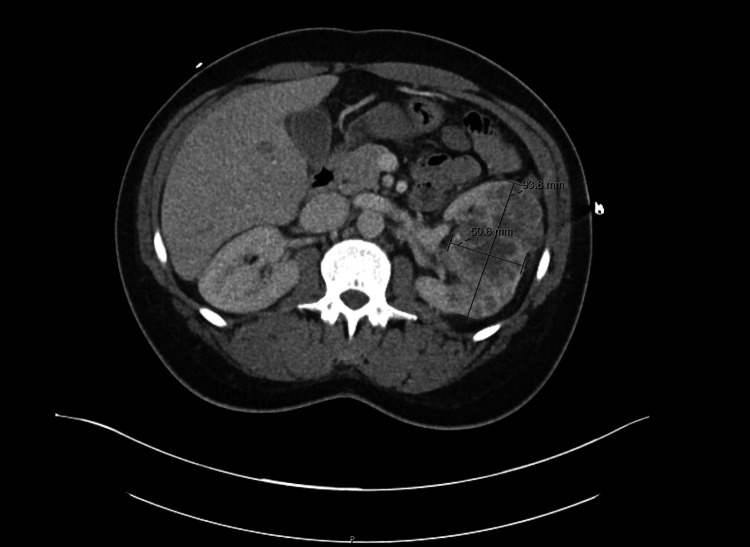
A CT scan of the abdomen shows a left renal mass

**Figure 3:**
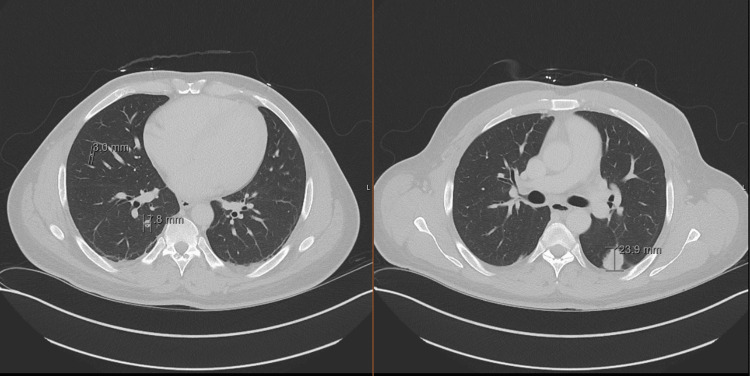
A CT scan of the chest shows the largest nodule in the left lung, measuring 23.9 mm, and multiple pulmonary nodules in the right lung

A computed tomography (CT) scan of the head showed no acute intracranial abnormalities. A CT scan of the chest, abdomen, and pelvis showed a large heterogeneous left renal mass measuring 9.4 x 5.1 x 10 cm in size, suspicious for primary renal neoplasm (Figure 2), and numerous pulmonary nodules, the largest being a 2.2 cm pleural-based nodule in the superior segment of the left lower lobe (Figure 3).

Magnetic Resonance Imaging (MRI) of the brain showed no evidence of intracranial mass, metastatic disease, or ischemic stroke. Because of these findings, the primary diagnosis of malignancy-associated TMA was made and TPE was not initiated.

Subsequently, he underwent an ultrasound-guided supraclavicular lymph node biopsy, which showed a reactive lymph node and was negative for malignancy. A few days later, ADAMTS13 activity was reportedly low at 3%, with an inhibitor level of 2 BEU (Bethesda Unit). This led to a diagnosis of immune-mediated TTP. However, the patient's platelet counts progressively improved over the next few days without intervention to 136000/microliter. His lactate dehydrogenase (LDH) also improved to 309 U/L. He was started on rituximab 375 mg/m2 weekly along with prednisone 1 mg/kg daily. He subsequently had a lung nodule biopsy before being discharged on an oral steroid taper with a plan to complete the remaining three cycles of rituximab infusions as an outpatient.

**Figure 4:**
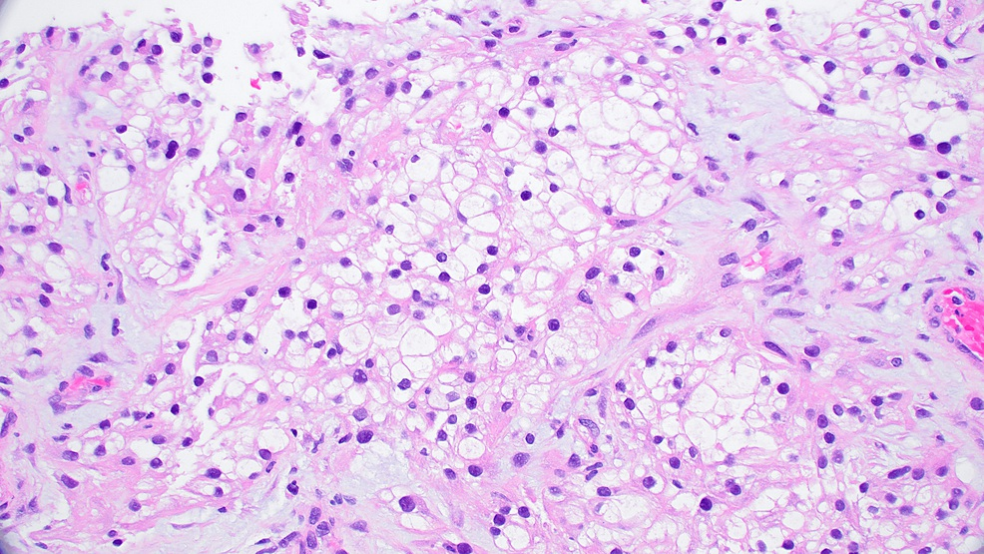
The core biopsy of the lung mass demonstrates neoplastic cells with clear cytoplasm and small hyperchromatic nuclei (A, H&E, 400x) H&E: hematoxylin and eosin-stained

**Figure 5:**
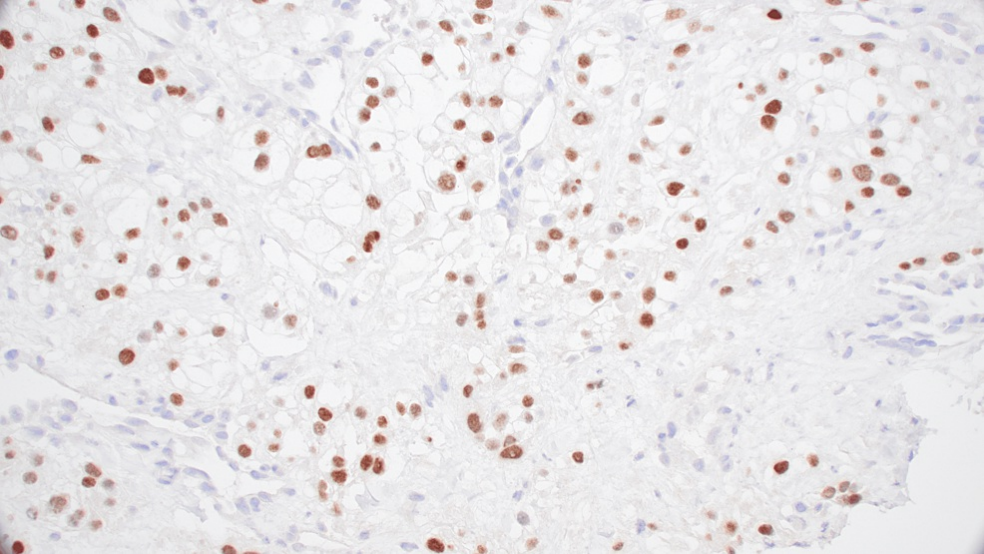
Immunohistochemical stain for carbonic anhydrase IX (CA IX) shows strong membranous staining compatible with clear cell renal cell carcinoma (C, 400x)

**Figure 6:**
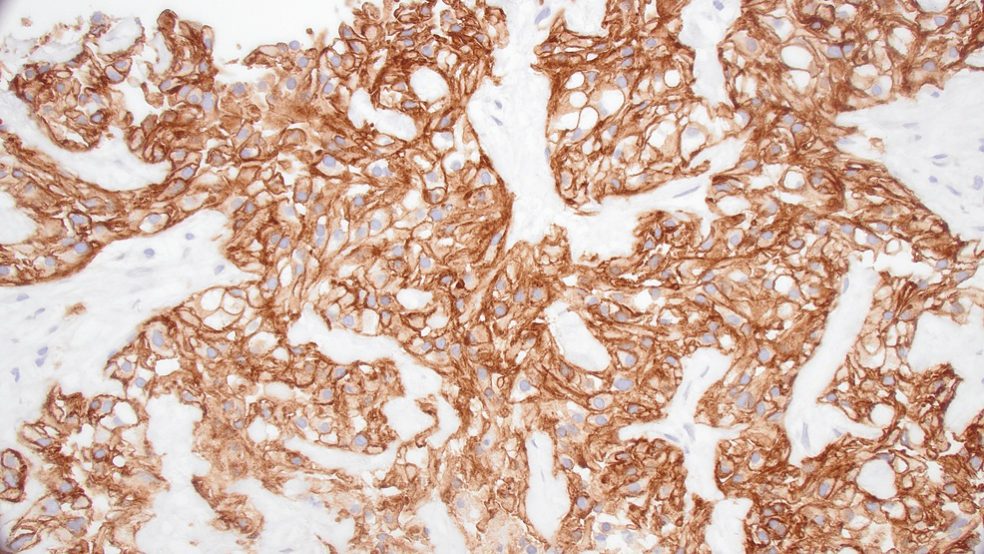
Immunohistochemical stain for paired-box gene 8 (PAX8) is positive and compatible with renal origin (B, 400x)

He was seen a week later in the oncology clinic for a follow-up and was found to have severe thrombocytopenia with a platelet count of 6000/microliter, LDH 700 U/L, and haptoglobin less than 7 milligrams per deciliter (mg/dl). He was directly admitted to the intensive care unit (ICU) and started on daily therapeutic plasma exchange (TPE). Rituximab infusion was continued along with methylprednisolone (1 gm daily for three days, followed by 1 mg/kg/day). A lung nodule biopsy done during his previous admission resulted in metastatic clear-cell renal cell carcinoma (Figures 4-6).

**Figure 7:**
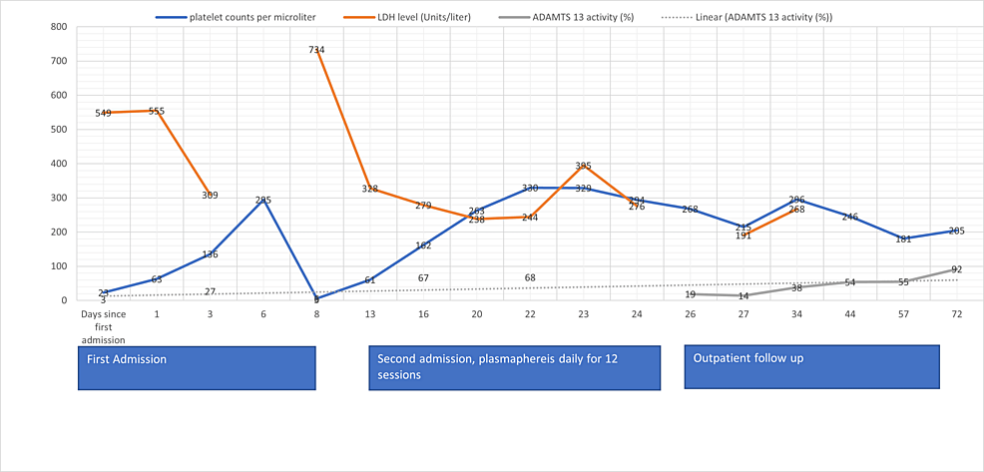
Trends of platelets, LDH, and ADAMTS13 since the first admission until the latest follow-up

A bone marrow biopsy was done due to the leukoerythroblastic reaction seen on the peripheral smear to rule out any bone marrow involvement by malignancy and showed hypercellular (70%) bone marrow with increased morphologically unremarkable trilineage hematopoiesis and no evidence of a hematolymphoid neoplasm. He received 12 sessions of plasma exchange (PLEX) therapy, and subsequently, his ADAMTS13 activity improved to 68%, with his platelet count improving to 268000/microliter (Figure 7).

He completed four cycles of weekly rituximab and was discharged on an oral prednisone taper. He was started on cabozantinib (60 mg oral daily) a week after discharge as an outpatient. Subsequent genetic testing did not reveal any significant mutations. He continues to follow up in the oncology clinic periodically as an outpatient. For the past six months, his ADAMTS 13 activity has persistently remained >50% after an initial drop to as low as 14% a week after discharge. Over the past six months, his platelet count has persistently remained >200,000/dl. Repeat CT of the chest, abdomen, and pelvis showed a stable renal mass and stable bilateral pulmonary nodules with no signs of cancer progression. He has been tolerating cabozantinib well.

## Discussion

TTP is a rare and life-threatening disorder distinguished by severe thrombocytopenia, microangiopathic hemolytic anemia, and multiorgan ischemia from widespread microvascular thrombi [5]. It is caused by a severe functional deficiency of ADAMTS13 (<10%), a specific von Willebrand factor-cleaving protease [6]. Patients with immune-mediated TTP usually have very low plasma levels of ADAMTS13, both during acute and disease-free periods. Rapid recognition of TTP is crucial to initiating appropriate treatment. A significant practical problem is the delay in ADAMTS13-level reporting. In severe cases of TTP, the cornerstone of management is therapeutic plasma exchange until the target platelet count is more than 100,000/mm3. For other non-emergent situations, high-dose steroid therapy and rituximab are proven effective therapies. Evidence supporting the use of TPE in the treatment of TMAs (other than TTP and TMA-complement-mediated) is lacking, and therefore its role is uncertain [7].

Thrombotic microangiopathy (TMA) due to malignancy has the potential to be misdiagnosed as TTP, with serious implications. In a systematic review by Francis et al., 10 out of 351 patients from one study and an additional 19 patients from the second study who had been initially diagnosed with TTP and subjected to directed treatment like plasmapheresis were subsequently found to have disseminated malignancy. The severity of anemia, thrombocytopenia, and neurologic and renal abnormalities was similar in patients with idiopathic TTP. Also, the patients with disseminated malignancy had poorer plasmapheresis responses and worse outcomes [8]. Most cancer-related TMAs have been reported in patients with mucin-producing adenocarcinomas such as gastric cancer (26.2%), breast cancer (21.4%), prostate cancer (13.7%), lung cancer (9.5%), and those with disseminated malignancy [9]. Multiple etiological hypotheses have been proposed for TMA, including abnormal angiogenesis in the marrow, aggressive growth of tumors, and secondary myelofibrosis causing endothelial cell lining injury of the marrow leading to the release of ultra-large VWF multimers [10]. Moreover, chemotherapy may also cause TMA by two mechanisms: an acute immune-mediated reaction or dose-dependent toxicity [10]. Maintaining a high clinical index of suspicion and paying attention to subtle differentiating features of these two identical conditions can help initiate prompt treatment and avoid complications from an unwarranted procedure. Presenting symptoms such as dyspnea, cough, pain other than abdominal pain, a history of cancer, and an extreme elevation of serum LDH level (attributed to tumor lysis) may suggest the presence of an occult malignancy. Similarly, many nucleated cells plus immature granulocytes may suggest underlying malignancy. Disseminated intravascular coagulation (DIC) is a common finding in patients with systemic malignancies. However, its absence does not exclude malignancy. ADAMTS13 activity in cancer-related TMA may be lower than normal (usually >20%) but is not severely deficient compared to TTP (<10%). Most importantly, failure to respond to plasmapheresis should cause concern for underlying cancer-related TMA. Bone marrow biopsy and immunohistochemistry, along with CT scans and bone scans alone or in combination, improve occult malignancy's diagnostic sensitivity in patients with TMA [11].

Our patient's profile and initial clinical presentation were highly suspicious for TTP. Acquired TTP predominantly affects the young population (median age 40) and has a high predilection for the black population [12]. Although his initial PLASMIC score was 5, placing him in the intermediate risk group, the concurrent diagnosis of RCC along with a spontaneous resolution of the symptoms initially led to the withholding of plasmapheresis, as cancer-induced TMA was considered the primary diagnosis. This decision was made after close consultation with a hematologist and transfusion specialist. For unclear reasons, his platelet count improved progressively over the next three days without any intervention, and he did not receive plasmapheresis during his first hospitalization. A few days later, ADAMTS13 levels were reported as very low. This led to a change in diagnosis to TTP, and he was started on rituximab infusions and high-dose steroids before discharge. This highlights the diagnostic and treatment challenges faced by patients with microangiopathic hemolytic anemia and thrombocytopenia with a concurrent metastatic cancer diagnosis.

The manifestation of TTP in the background of a newly diagnosed RCC in our patient is quite peculiar. It is unclear whether RCC has any causal relationship with the TTP seen in our patient. RCC is known as an "immunogenic tumor" based mainly on the positive response of IL-2 or interferon-alpha interferon- α (IFNα) on the tumor. For non-clear-cell RCC variants, response to checkpoint inhibitors suggests a dominant immune component in them as well [13]. More commonly, immune-mediated ITP has been reported in a handful of cases of RCC [14, 15]. However, to our knowledge, to date, there has been no report of TTP in a treatment-naive RCC patient.

The therapeutic challenges after diagnosing immune-mediated TTP in a patient with RCC extend beyond the use of TPE, steroids, or rituximab. The National Comprehensive Cancer Network (NCCN) guidelines recommend stratified treatments by histology and also by risk group. Preferred first-line therapy for all risk groups is axitinib (a selective, second-generation tyrosine kinase inhibitor (TKIs)of vascular endothelial growth factor receptors (VEGFRs)) with pembrolizumab (a monoclonal antibody that selectively binds to programmed death-1 (PD-1; expressed on activated T cells) and blocks the interaction between PD-1 and its ligands, programmed death ligand 1 (PD-L1) and PD-L2 (both expressed on antigen-presenting cells). Similarly, cabozantinib (a multitargeted TKI of VEGFRs, mesenchymal-epithelial transition (MET), and AXL receptor tyrosine kinase) with nivolumab (an anti-PD-1 antibody) is recommended as first-line therapy for clear-cell RCC in all risk group patients (category 1). Lenvatinib (a multitargeted TKI of VEGFR-1, -2, and -3; fibroblast growth factor receptor-1, -2, -3, and 4; platelet-derived growth factor receptor-α (PDGFR-α); c-KIT; and RET) with pembrolizumab is another recommended first-line therapy for clear-cell RCC across all risk groups. For poor or intermediate risk groups, NCCN guidelines suggest ipilimumab with nivolumab as category 1 and cabozantinib as category 2A recommendations. [16] However, due to immune-mediated TTP, our patient was not considered an ideal candidate for any immunotherapy. He was also deemed an inappropriate candidate for participation in clinical trials due to the immune-mediated TTP, as most of the ongoing trials were immunotherapy-based. He was subsequently started on cabozantinib (a tyrosine kinase inhibitor), which he is tolerating well with a good response.

## Conclusions

TTP is a very rare but life-threatening condition that needs urgent diagnosis and treatment. TMAs due to metastatic malignancies can often be misdiagnosed as TTPs with serious implications. Concurrent presentation of TTP with metastatic cancer can add to diagnostic and therapeutic challenges. ADAMTS13 activity can be helpful in differentiating these two similar yet different clinical conditions. It is important to maintain a high clinical index of suspicion and pay attention to subtle yet important differentiating features of these two similar yet different clinical conditions for prompt initiation of appropriate treatment and to avoid unwarranted complications. The manifestation of TTP in a newly diagnosed RCC, like in our patient, has therapeutic challenges, including making a TPE decision and the choice of treatment for the cancer itself.
